# Guidance for Canadian Breast Cancer Practice: National Consensus Recommendations for the Systemic Treatment of Patients with HER2+ Breast Cancer in Both the Early and Metastatic Settings (2025 Update)

**DOI:** 10.3390/curroncol33040200

**Published:** 2026-03-31

**Authors:** Aalok Kumar, Katarzyna J. Jerzak, Karen A. Gelmon, Jean-François Boileau, Nathaniel Bouganim, Christine Brezden-Masley, Jeffrey Q. Cao, David W. Cescon, Stephen Chia, Scott Edwards, Anil Abraham Joy, Kara Laing, Nathalie LeVasseur, Sandeep Sehdev, Christine Simmons, Marc Webster, Mita Manna, on behalf of Patient Advocacy, Breast Cancer Canada

**Affiliations:** 1 BC Cancer—Surrey, University of British Columbia, Vancouver, BC V3V 1Z2, Canada; akumar7@bccancer.bc.ca; 2 Sunnybrook Odette Cancer Centre, University of Toronto, Toronto, ON M4N 3M5, Canada; katarzyna.jerzak@sunnybrook.ca; 3 BC Cancer—Vancouver, University of British Columbia, Vancouver, BC V5Z 4E6, Canada; 4 Jewish General Hospital, McGill University, Montréal, QC H3T 1E2, Canada; 5 McGill University Health Centre, McGill University, Montréal, QC H4A 3J1, Canada; 6 Marvelle Koffler Breast Centre, Mount Sinai Hospital, University of Toronto, Toronto, ON M5G 1X5, Canada; 7 Arthur J.E. Child Cancer Centre, University of Calgary, Calgary, AB T2N 5G2, Canada; jeffrey.cao@albertahealthservices.ca (J.Q.C.);; 8 Princess Margaret Cancer Centre, University of Toronto, Toronto, ON M5G 2M9, Canada; 9 Dr. H. Bliss Murphy Cancer Centre, Memorial University of Newfoundland, St. John’s, NL A1B 3V6, Canada; 10 Cross Cancer Institute, University of Alberta, Edmonton, AB T6G 1Z2, Canada; 11 The Ottawa Hospital Cancer Centre, Ottawa, ON K1H 8L6, Canada; 12 Saskatoon Cancer Centre, University of Saskatchewan, Saskatoon, SK S7N 4H4, Canada

**Keywords:** antibody–drug conjugate, breast cancer, HER2-positive, HER2+, HER2-directed therapy, REAL Alliance

## Abstract

Human epidermal growth factor receptor 2-positive (HER2+) breast cancer is an aggressive subtype associated with a poor prognosis when not optimally treated. Over the past year, major advances—including results from DESTINY-Breast05, DESTINY-Breast09, DESTINY-Breast11, PATINA, and long-term APHINITY follow-up—have changed the treatment landscape regarding the place in therapy of antibody–drug conjugates and the optimal sequencing of systemic therapies. These developments prompted the need for updated evidence-informed consensus recommendations to support consistent, high-quality care across Canada. Research Excellence, Active Leadership Canadian Breast Cancer Alliance (REAL Alliance), comprising clinical-academic oncologists from across Canada and Breast Cancer Canada, updated its 2024 HER2+ recommendations through a modified Delphi process with up to three rounds of anonymous voting. Consensus was defined a priori as ≥75% agreement. This 2025 update incorporates new data in early-stage, metastatic, and central nervous system-involved disease, including revisions to neoadjuvant and adjuvant treatment pathways and expanded guidance on the clinical use of antibody–drug conjugates.

## Simple Summary

HER2-positive (HER2+) breast cancer is a fast-growing type of breast cancer that can have poor outcomes if not treated effectively. Over the past year, results from many important clinical trials were released, leading to improvements in how this cancer is treated. Newer treatments, including antibody–drug conjugates, have contributed significant improvements in both early and advanced settings. Because of these changes, Research Excellence, Active Leadership Canadian Breast Cancer Alliance (REAL Alliance) updated its HER2+ treatment recommendations using a structured expert-consensus approach. The 2025 update reflects new evidence for HER2+ early-stage breast cancer, advanced (metastatic) disease, and cancer that has spread to the brain. It includes updated guidance on treatments given before and after surgery, as well as expanded recommendations on when and how to use antibody–drug conjugates in clinical practice.

## 1. Introduction

As noted in our previous publications [1,2] and restated here, Research Excellence, Active Leadership Canadian Breast Cancer Alliance (REAL Alliance) is an equitable standing nucleus committee of multidisciplinary, clinical-academic oncologists and other specialists in breast cancer from across Canada and representatives from Breast Cancer Canada, a patient advocacy organization. Formed in December 2023, it provides a national ecosystem of leadership to develop evidence-informed guidance and recommendations for equitable breast cancer clinical management. REAL Alliance publishes national clinical consensus recommendations to promote timely health policy, funding, and consistent clinical adoption based on research evidence and clinical expertise to ensure optimal outcomes for breast cancer patients across all provinces and territories in Canada.

Building on prior publications in this series, the present manuscript provides guidance for the systemic treatment of human epidermal growth factor receptor 2-positive (HER2+) breast cancer, offering a framework that integrates tumour biology, neoadjuvant treatment, pathologic response, adjuvant treatment, and patient preferences into shared decision-making.

## 2. Materials and Methods

### 2.1. Consensus Recommendation Process

For the purposes of this update, we reviewed peer-reviewed publications from January to October 2025 and major conference presentations from San Antonio Breast Cancer Symposium (SABCS) 2024, American Society of Clinical Oncology (ASCO) 2025, and European Society for Medical Oncology (ESMO) 2025, focusing on phase II/III trials with potential to affect clinical practice. Four members of REAL Alliance (A.K., K.J.J., K.A.G., M.M.) acted as the sub-committee and identified the most relevant evidence based on clinical applicability and methodological rigour. The sub-committee also reviewed the 2024 recommendations and identified and revised those affected by new evidence. The updated statements were subjected to a modified Delphi process of anonymous voting by an expert panel using an electronic platform. The panel consisted of 13 medical oncologists, 1 surgical oncologist, 1 radiation oncologist, 1 oncology pharmacist, and 1 patient advocacy representative, all with specialized expertise and extensive experience in managing breast cancer. The patient advocacy representative from Breast Cancer Canada participated as a full voting member of the panel and contributed to discussion and recommendation development to ensure incorporation of patient perspectives. For the purposes of the voting document, all statements were included, even those not updated, and voters were given the opportunity to comment on all statements. The consensus voting results are presented in Table S1.

A recommendation was considered accepted if 75% or more of the participants agreed with it after the predefined rounds of voting. The strength of recommendations was defined as follows for the purposes of the 2025 publications: a strong recommendation is based on the highest quality of evidence; a moderate recommendation (termed strong consideration in the 2024 publications) is supported by evidence that is less robust, indirect, or limited in scope; a weak recommendation (termed moderate recommendation in the 2024 publications) is based on lower quality evidence; and an expert opinion reflects recommendations where there is endorsement by REAL Alliance, but for which there is limited evidence. As a final step, the sub-committee compared the current ESMO and ASCO guidelines to REAL consensus recommendations.

### 2.2. Guiding Principles

The guiding principles are outlined in our 2024 publication [1] and are summarized and updated here. Given the biology of breast cancer, the most effective therapies should be used across both early and advanced disease settings. Timely initiation of therapy is critical. Canadian Partnership Against Cancer states that “*initial treatment (surgery, or systemic therapy and/or radiation therapy started) (…) should be initiated within 4 weeks of consultation. Appropriate referrals should be made as early as possible*” [3].

In HER2+ breast cancer (and triple-negative breast cancer), early multidisciplinary assessment is essential to ensure that neoadjuvant treatment is considered where appropriate. In early HER2+ breast cancer, REAL Alliance recognizes the changing neoadjuvant landscape that may warrant escalation or de-escalation of adjuvant therapy based on surgical outcomes.

Although recommendations for advanced disease are organized by line of therapy, treatment decisions are intended to be guided by disease biology and individual patient factors, including comorbidities, performance status, the presence of brain metastases, and patient preferences, rather than rigid sequencing. Shared decision-making is integral to all treatment decisions and is emphasized when the treatment choice is not straightforward.

Comprehensive molecular profiling is increasingly integrated into metastatic breast cancer management. As outlined in our HR+/HER2− metastatic guidance, testing for somatic pathway alterations (e.g., PIK3CA, PTEN, AKT, ESR1) and pathogenic germline variants should be performed when results may inform treatment decisions [4]. In HER2+ disease, biologic factors such as intratumoural HER2 heterogeneity may influence response to HER2-directed therapies. However, at this time, prospective data supporting routine biomarker-directed modifications of HER2-targeted treatment sequencing based on heterogeneity is lacking.

Special populations, including older adults and patients with significant comorbidities or organ dysfunction, require individualized treatment selection. Chronological age alone should not preclude evidence-based therapy, though comprehensive assessment of frailty, performance status, and organ function is essential when selecting and sequencing systemic treatments. Dose modifications and organ-specific adjustments should follow product monograph recommendations and institutional protocols.

With the expanding use of antibody–drug conjugates (ADCs) across both early and advanced disease settings, clinicians should be attentive to their distinct toxicity profiles, including hematologic, pulmonary, hepatic, gastrointestinal, and ophthalmologic effects. Careful baseline assessment of performance status, frailty, organ function, and comorbidities is essential when selecting and sequencing these therapies.

The recommendations are based on the best available evidence at the time, with the goal of optimizing outcomes in Canadian practice. They are developed independent of reimbursement considerations and may precede regulatory approval or health-system adoption.

When a recommended therapy is pending Health Canada approval, clinicians should continue to optimize approved standards of care consistent with current practice until regulatory approval is granted. During this interim period, clinicians may also consider referral for clinical trial participation where feasible and appropriate. Cancer centres may wish to anticipate operational readiness to include standardized symptom assessments, toxicity monitoring protocols, multidisciplinary coordination, and patient education so that evidence-supported therapies can be implemented safely and efficiently once approved.

Canada’s decentralized funding environment may result in differences in the timing of adoption across provinces and territories, with potential inequities in access. While awaiting public reimbursement, clinicians can establish referral pathways for clinical trial availability, document clinical rationale based on evidence, and engage in multidisciplinary teams early to reduce delays once treatments are available.

As always, enrollment in clinical trials is encouraged whenever patients meet eligibility criteria.

## 3. Systemic Therapy in HER2+ Early Breast Cancer

### 3.1. Early Breast Cancer

The 2025 updates included in the early setting are itemized below. Narrative on pathologic complete response (pCR) is included as a preamble to the recommendations, as it is particularly relevant in HER2+ breast cancer and is widely considered a surrogate endpoint reflective of event-free survival (EFS) or overall survival (OS) benefit [5]. Recommendation 2 has not changed but is included in this update to address the clinical controversy with cT1cN0 tumours. Recommendation 8 has been significantly revised based on data presented at ESMO 2025. Recommendations 5 and 6 have additional evidence added to the narrative.

The REAL Alliance stated the following in our 2024 publication [1]: “*For those patients who receive neoadjuvant therapy, pCR—the complete elimination of invasive disease in the breast and axillary lymph nodes—is an important intermediate outcome for early-stage HER2+ breast cancer. Experiencing pCR (or not) has strong implications for prognosis and subsequent adjuvant treatment. Patients who experience pCR have significantly improved long-term disease-free survival (DFS) compared to those harbouring residual disease, although the extent of residual disease also affects outcome. Indeed, residual invasive disease is the most significant adverse prognostic factor for DFS and OS in HER2+ breast cancer [5,6,7,8]; thus, it is important to identify these patients and escalate systemic therapy, as described in Recommendation 8. International efforts are being made to identify treatments that optimize pCR rates to improve the potential for long-term cure [7]. Notably, the importance of pCR and treatments that improve pCR rates have been incorporated into multiple European and American treatment and funding guidelines [1,7,9].*” This bears repeating, as the pCR results of DESTINY-Breast11 were presented at ESMO 2025. The significance of these results is further discussed in Recommendation 5.

Table 1 summarizes the REAL Alliance recommendations for patients with HER2+ early breast cancer and compares these to ESMO and ASCO guidelines.

**Recommendation 2:**

*For patients with HER2+ early breast cancer cT1c (i.e., >1 to ≤2 cm) without evidence of nodal disease (cN0):*

*(a)*  *The standard of care is surgery followed by adjuvant treatment. [Strong recommendation.]*
*(b)*  *However, due to current global practices, consideration can be given to neoadjuvant treatment followed by surgery and adjuvant treatment. [Moderate recommendation.]*

Although the recommendation has not changed in this update, it is important to address the controversy with the cT1cN0 subgroup. Optimal treatment selection and sequencing of therapy is unclear. These tumours bridge low-risk disease suitable for de-escalated adjuvant taxane + trastuzumab (TH), as per the APT trial [12], and higher-risk stage II disease, where neoadjuvant therapy may be favoured. Upfront surgery risks undertreatment if nodal upstaging (pN^+^) is identified, as treatment escalation to adjuvant trastuzumab emtansine (T-DM1) requires that patients have received neoadjuvant treatment. A neoadjuvant strategy allows assessment of treatment response and enables escalation to T-DM1 for those with residual invasive disease at surgery, as established in the KATHERINE trial [13,14]. Also of note, several neoadjuvant chemotherapy regimens are available for HER2+ disease, including anthracycline + cyclophosphamide (AC), followed by TH (AC-TH) or taxane + carboplatin + trastuzumab (TCH). These combinations are well-established standards and provide robust pCR data [15]. Recently, HELEN-006, a phase III trial conducted in Asian patients, demonstrated that significantly more patients experienced a pCR with de-escalated neoadjuvant nab-paclitaxel + trastuzumab + pertuzumab (HP) compared to docetaxel + carboplatin + HP (combined odds ratio 1.54; 95% confidence interval [CI] 1.10–2.14; stratified *p* = 0.011), with fewer grade 3–4 adverse events [16]. Weekly TH, as studied in the APT trial, is primarily an adjuvant de-escalation regimen and is not routinely used in the neoadjuvant setting; however, it may be considered in highly selected, lower-risk cases when chemotherapy intensity must be minimized. Treatment decisions for cT1cN0 disease should therefore be individualized through multidisciplinary discussion, balancing disease biology, patient comorbidities, and the potential implications of nodal upstaging.

**Recommendation 5:**

*For patients with HER2+ early breast cancer with ≥cT2 or those with nodal disease (cN+), the standard of care is neoadjuvant therapy. Treatment options are:*

*(a)*  *Trastuzumab + pertuzumab + chemotherapy (taxane backbone preferred) [Strong recommendation];*
*(b)*  For those meeting DESTINY-Breast11 criteria *, T-DXd ^†^ followed by trastuzumab + pertuzumab + taxane. [Moderate recommendation.]

* cT3 and cN0–3 or cT and cN1–3

*^†^* *Pending Health Canada approval*

The intended outcome of neoadjuvant therapy is pCR, defined as the absence of residual invasive cancer in the breast and lymph nodes at surgery, and is one of the most important prognostic endpoints [5]. pCR not only helps avoid the morbidity of axillary lymph node dissection but is also a clinically validated surrogate for long-term outcomes in HER2+ disease, including EFS and OS [5,17]. In practical terms, patients whose disease achieves a pCR have substantially fewer recurrences and deaths, making pCR a meaningful short-term treatment goal associated with favourable long-term outcomes.

The standard of care for patients with HER2+ early breast cancer with ≥cT2 or node-positive (cN^+^) disease is neoadjuvant trastuzumab + pertuzumab + chemotherapy (THP), supported by pivotal studies such as NeoSphere, PEONY, TRYPHAENA, and TRAIN-2 [1,18,19,20,21]. These trials consistently demonstrated superior pCR and EFS with dual HER2 blockade compared with trastuzumab alone, along with meaningful nodal downstaging and reduced surgical morbidity.

Recently, the phase III DESTINY-Breast11 (DB11) trial, presented at ESMO 2025, investigated a neoadjuvant approach for patients with higher-risk disease, adding to the evolving landscape [22]. DB11 assessed the efficacy and safety of neoadjuvant trastuzumab deruxtecan (T-DXd) for four cycles followed by THP (T-DXd-THP) compared to dose-dense doxorubicin + cyclophosphamide (ddAC), followed by THP (ddAC-THP). T-DXd-THP achieved a pCR of 67.3% versus (vs.) 56.3% with ddAC-THP (absolute Δ 11.2%; *p* = 0.003), with fewer grade 3–4 adverse events (37.5% vs. 55.8%) and a comparable incidence of low-grade interstitial lung disease (ILD) (~5%). An adverse event leading to surgical delay occurred in 3.4% vs. 2.6% of patients in the T-DXd-THP arm and ddAC-THP arm, respectively. Regarding the use of T-DXd, the reader is referred to the Canadian product monograph for warnings/precautions and dosage/administration for guidance on the identification and management of ILD [23].

Notably, DB11 enrolled a higher-risk population than the trials mentioned above (NeoSphere, PEONY, TRYPHAENA, and TRAIN-2). Eligible patients for DB11 included those with previously untreated, locally advanced or inflammatory HER2+ breast cancer, including tumours ≥ cT3 irrespective of nodal status, or any primary tumour size accompanied by clinically positive lymph nodes. DB11 represents the first phase III evidence demonstrating the superiority of an ADC-based, non-anthracycline regimen over a conventional chemotherapy-based neoadjuvant strategy in HER2+ early breast cancer. However, while ddAC-THP served as the control regimen in DB11, anthracycline-free combinations such as taxane + carboplatin + HP (TCHP) are widely used, particularly for patients at increased risk of cardiac toxicity. TCHP was evaluated in the KRISTINE trial [24,25]. DB11 does not directly address whether T-DXd-THP is superior to this common TCHP regimen. While pCR is a validated prognostic surrogate associated with long-term outcomes in HER2+ early breast cancer, EFS data remain immature, with ~5% of prespecified events observed to date. Longer follow-up is required to confirm the durability of the benefit and to determine whether this approach should supplant established neoadjuvant THP regimens, including anthracycline-free options commonly used in practice.

For patients whose disease achieved a pCR, DB11 allowed for trastuzumab +/− pertuzumab for 1 year. For patients with residual invasive disease, DB11 mandated T-DM1 for 14 cycles. It is noteworthy that only 53% and 57% of patients with residual invasive disease received T-DM1 in the T-DXd-THP arm and ddAC-THP arm, respectively.

Accordingly, for patients with HER2+ early breast cancer ≥cT2 or cN^+^ disease, neoadjuvant therapy remains the standard of care, with pCR at surgery serving as an important prognostic endpoint and measure of treatment response. Treatment options include trastuzumab + pertuzumab combined with chemotherapy, typically a taxane backbone, with anthracycline-containing or anthracycline-free regimens selected according to patient and disease factors. For patients with higher-risk disease meeting DB11 criteria, T-DXd-THP is a treatment option, pending Health Canada approval. Until such time as Health Canada approval is granted, clinicians should continue to use approved neoadjuvant regimens consistent with current standards of care.

**Recommendation 6:**

*Although neoadjuvant treatment is preferred, for those patients who are treated with upfront surgery and are then found to have nodal disease in the pathological specimen (pN+), the standard of care is adjuvant trastuzumab + chemotherapy followed by trastuzumab, with consideration given to the addition of pertuzumab for a total of 1 year. [Moderate recommendation.]*

This recommendation is unchanged from our 2024 guidance and, as noted in our 2024 publication [1] and restated here, is intended to inform clinical decision-making specifically for patients in whom nodal involvement is identified only after surgery on pathological review. It is based on the APHINITY trial, which was recently updated at the ESMO Breast Cancer 2025 conference [26].

The APHINITY 10-year update demonstrated an OS improvement from 89.8% to 91.6% with the addition of pertuzumab to trastuzumab in the adjuvant setting (hazard ratio [HR] 0.83, 95% CI 0.69–1.00; *p* = 0.044) and a 21% relative reduction in risk of death among patients with node-positive disease (HR 0.79, 95% CI 0.64–0.97) [26]. The invasive DFS advantage persisted (HR 0.79, 95% CI 0.68–0.92), with no new cardiac or treatment-related toxicities. These data confirm a durable but modest absolute OS gain (~1.8%) and excellent tolerability, supporting the addition of adjuvant pertuzumab as a reasonable intensification strategy when pN^+^ disease is discovered postoperatively.

Despite this positive update, and although some panel members advocated for a strong recommendation, REAL Alliance, following careful deliberation, maintained this recommendation as moderate. The decision reflected the group’s emphasis on prioritising neoadjuvant therapy whenever feasible. While adjuvant HP is appropriate for patients who did not receive neoadjuvant therapy, neoadjuvant THP maximizes therapeutic options, including response-based escalation to T-DM1. Early referral for neoadjuvant therapy in clinically ≥cT2 or cN^+^ disease remains essential.

**Recommendation 8:**

*For patients with HER2+ early breast cancer in whom residual invasive disease is detected pathologically in the surgical specimen of the breast or axillary lymph nodes after completion of neoadjuvant trastuzumab + pertuzumab + chemotherapy, the standard of care is escalation of adjuvant therapy. Treatment options include:*

*(a)*  For disease not meeting high-risk criteria *, adjuvant therapy with T-DM1 for 14 cycles. [Strong recommendation.]
*(b)*  For high-risk disease *, adjuvant therapy with T-DXd ^†^ for 14 cycles, replacing T-DM1. [Strong recommendation.]

* High-risk criteria: inoperable early breast cancer (cT4, N0–3 or cT1–3, N2–3) with any residual disease or operable early breast cancer (cT1–3, N0–1) with residual axillary node-positive disease (ypN1–3) after neoadjuvant chemotherapy.

^†^ Pending Health Canada approval

The phase III DESTINY-Breast05 (DB05) trial presented at ESMO 2025 [27] has redefined the post-neoadjuvant treatment landscape for patients with HER2+ early breast cancer who have residual invasive disease following neoadjuvant THP. In this study, 1635 patients with high-risk HER2+ early breast cancer (i.e., defined as inoperable disease at presentation with any residual disease after neoadjuvant therapy, or operable disease with residual nodal involvement) were randomly assigned to receive T-DXd or T-DM1, each administered for 14 cycles in the adjuvant setting.

DB05 demonstrated a statistically significant and clinically meaningful improvement in invasive DFS with T-DXd compared with T-DM1, achieving an 8.7% absolute improvement in 3-year invasive DFS (92.4% vs. 83.7%) and a 53% reduction in risk of invasive recurrence or death (HR 0.47, 95% CI 0.34–0.66; *p* < 0.0001). A longer follow-up is required to determine OS, as, currently, only T-DM1 has proven OS benefit [13].

Notably, the incidence of central nervous system (CNS) events was numerically lower in the T-DXd arm, although event rates remain low and additional follow-up is required to confirm this finding. The safety profile of T-DXd was consistent with prior experience in the metastatic setting, with ILD observed in ~9.6% of patients compared with 1.6% in the T-DM1 arm, primarily grade 1–2 events. These data underscore the importance of institutional readiness for ILD monitoring, including standardized symptom assessment and imaging protocols, early intervention pathways, and appropriate antiemetic prophylaxis in routine practice.

Taken together, these findings represent the first clear demonstration of superior efficacy for a novel ADC over T-DM1 in the adjuvant setting, establishing T-DXd as a new standard of care for patients with high-risk HER2+ early breast cancer and residual invasive disease after neoadjuvant therapy. While T-DXd delivers unprecedented benefit in this population, it is essential to note that DB05 enrolled a distinctly high-risk cohort—patients with inoperable disease at presentation or residual nodal involvement after neoadjuvant therapy. Accordingly, these findings should not be extrapolated to patients with minimal residual disease who do not meet DB05 high-risk criteria, in whom T-DM1 remains the evidence-based standard. Specifically, patients who were operable at presentation (cT1–3N0–1) and have residual invasive disease confined to the breast without residual nodal involvement (ypN0) do not meet DB05 high-risk criteria and would receive adjuvant T-DM1, as established in the KATHERINE trial.

Until Health Canada approval for DB05 is granted, clinicians should continue to use approved adjuvant escalation strategies consistent with current standards of care.

For patients who do not meet these high-risk criteria and who have residual invasive disease at surgery after neoadjuvant treatment, T-DM1 × 14 cycles in the adjuvant setting is the preferred treatment as established by the KATHERINE trial, where invasive DFS was significantly higher with adjuvant T-DM1 vs. trastuzumab at 3-year follow-up (88.3% vs. 77.0%; HR 0.50, 95% CI 0.39–0.64; *p* < 0.001) [14]. The KATHERINE trial allowed neoadjuvant chemotherapy reflective of real-world practice (i.e., any standard neoadjuvant chemotherapy containing a taxane and trastuzumab +/− anthracyclines, carboplatin, or pertuzumab).

**Figure 1:**
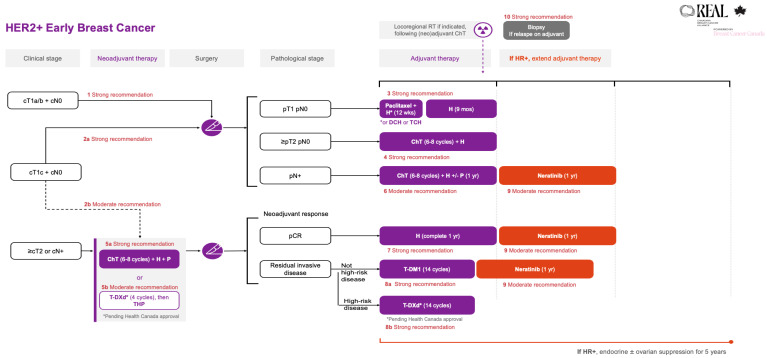
REAL Alliance recommendations for the treatment of HER2+ early breast cancer. ChT, chemotherapy; cN0, no nodal disease based on clinical assessment; cN+, nodal disease based on clinical assessment; cT1a/b, tumour ≤ 1 cm on clinical assessment; cT1c, tumours > 1 cm, but ≤2 cm on clinical assessment; cT2, tumours > 2 cm but ≤5 cm on clinical assessment; DCH, docetaxel + cyclophosphamide + trastuzumab; H, trastuzumab; HR+, hormone receptor positive; p, based on pathologic assessment; P, pertuzumab; pCR, pathologic complete response; RT, radiotherapy; T-DM1, trastuzumab emtansine; T-DXd, trastuzumab deruxtecan; TCH, docetaxel + carboplatin + trastuzumab; THP, taxane + trastuzumab + pertuzumab.

All the REAL Alliance recommendations for HER2+ early breast cancer are outlined in an algorithm (see Figure 1).

### 3.2. Metastatic Breast Cancer

Updates in the metastatic setting include additional treatment options in the first-line setting, as noted in Recommendation 11, as well as additional guidance on sequencing in subsequent lines of therapy (Recommendations 13 and 14). The REAL Alliance recommendations for metastatic breast cancer are summarized in Table 2 and compared with ESMO and ASCO guidelines.

**Recommendation 11:**

*For patients with HER2+ (HR±) metastatic breast cancer:*

- Who have not received prior HER2-directed therapy or chemotherapy (de novo);
- Whose disease relapses >6 months after completion of (neo)adjuvant chemotherapy + HER2-directed therapy (late relapse), treatment options include:

*(a)*  *Trastuzumab + pertuzumab + taxane chemotherapy followed by trastuzumab + pertuzumab +/− ET maintenance therapy, the current standard of care. [Strong recommendation.]*
*(b)*  T-DXd * + pertuzumab could be considered with shared decision-making *. [Moderate recommendation.]*

* Pending Health Canada approval

The standard of care for this patient population, as noted in our 2024 publication and 11a above, is trastuzumab + pertuzumab + taxane chemotherapy, followed by maintenance trastuzumab + pertuzumab +/− ET [1].

The DESTINY-Breast09 (DB09) study [32], presented at ESMO 2025, has sparked significant discussion regarding first-line therapy in HER2+ metastatic breast cancer. In this large phase III trial, patients with either de novo metastatic disease or those whose disease relapsed more than six months after (neo)adjuvant therapy were randomized to receive T-DXd +/− pertuzumab or the standard CLEOPATRA regimen [33] of THP. The prespecified interim analysis reported data for the T-DXd + pertuzumab group and the THP group. Results showed a 14-month gain in median progression-free survival (PFS) with T-DXd + pertuzumab compared with THP, a magnitude of benefit not previously seen in this setting. Response rates exceeded 80%, and efficacy appeared consistent across key subgroups, including hormone-receptor status, prior exposure to HER2-directed therapy, and *PIK3CA* mutation status.

These data are from the preplanned interim analysis, with OS results and outcomes from the T-DXd monotherapy arm still pending. Also, the median duration of T-DXd of ~20 months raises important questions about the sustainability of long-term therapy and the cumulative burden of toxicity, particularly nausea and ILD. This underscores the importance of shared decision-making, weighing the potential for prolonged disease control against treatment intensity and quality-of-life considerations. Importantly, patients receiving frontline T-DXd + pertuzumab should be carefully monitored to ensure ongoing eligibility for T-DM1 in subsequent lines should relapse occur early or toxicity necessitate treatment discontinuation.

At present, T-DXd + pertuzumab represents a treatment option. However, until mature data are available, THP should remain the preferred first-line regimen. The rapid evolution of the HER2+ metastatic landscape underscores the need for individualized treatment sequencing and ongoing reassessment and updating of these guidance documents as new evidence emerges.

Just prior to publication, the results of HER2CLIMB-05 were presented at SABCS 2025, which demonstrated a statistically significant improvement in PFS with the addition of tucatinib to first-line maintenance therapy for HER2+ metastatic disease (HR 0.641, 95% CI 0.514–0.799; *p* < 0.0001; median PFS: 24.9 vs. 16.3 months) [34].

*(c)*  *For triple-positive disease only, trastuzumab + pertuzumab + taxane chemotherapy, followed by maintenance palbociclib + endocrine therapy (aromatase inhibitor or fulvestrant ± ovarian suppression) + trastuzumab + pertuzumab, could be considered. [Moderate recommendation.]*

The treatment of hormone receptor-positive (HR+)/HER2+ (triple-positive) metastatic breast cancer is also evolving with the inclusion of endocrine-targeted therapies into HER2-targeted regimens. While HER2 remains the principal disease driver in this biologic subtype, co-targeting the estrogen receptor pathway is an option to extend disease control after initial chemotherapy-based induction in patients with triple-positive disease.

The phase III PATINA trial (AFT-38) evaluated the addition of palbociclib to maintenance HER2-directed therapy (HP) combined with endocrine therapy (ET), as either an aromatase inhibitor or fulvestrant, with ovarian function suppression, in patients with HR+/HER2+ metastatic breast cancer who had achieved at least stable disease following induction with THP [35]. At SABCS 2024, investigators reported a 15.2-month improvement in median PFS with the addition of palbociclib (44.3 months vs. 29.1 months; HR 0.74, 95% CI 0.58–0.94), representing one of the longest PFS durations observed in this subtype [36]. Toxicity was manageable and consistent with known cyclin-dependent kinase 4 and 6 (CDK4/6) inhibitor-related effects, with no new safety signals. Importantly, health-related quality-of-life analyses presented at ESMO 2025 [37] demonstrated stability across global and functional scales and no clinically meaningful deterioration compared with control, reinforcing the tolerability of long-term palbociclib-based maintenance.

These results support the use of CDK4/6 inhibitor-based maintenance therapy following THP induction in appropriately selected patients. No phase III data exists for other CDK4/6 inhibitors in this setting. Thus, palbociclib remains the only agent with prospective evidence supporting its use alongside HER2-targeted maintenance therapy.

While the addition of palbociclib to HP + ET maintenance can be considered for fit, motivated patients after discussion of potential benefits and risks, the established standard first-line approach remains THP followed by HP ± ET maintenance. As further maturation and peer-reviewed publication of the PATINA results emerge and as data from related studies explore alternative CDK4/6 inhibitors, REAL Alliance will continue to reassess the strength of this recommendation and its potential incorporation into routine practice.

**Recommendation 13:**

(a) *For patients with HER2+ metastatic breast cancer whose disease has progressed on first-line HER2-directed therapy that was not T-DXd, the standard of care is T-DXd, in the absence of contraindications. [Strong recommendation.]*

As described in Recommendation 11b, DB09 has established T-DXd as a treatment option in the first-line setting [32,38]. However, if a patient has not received T-DXd in the first-line setting, it should be considered upon disease progression.

(b) *For patients with HER2+ metastatic breast cancer whose disease has progressed following T-DXd, treatment options include tucatinib + capecitabine + trastuzumab (preferred), or T-DM1, or chemotherapy + HER2-directed antibody therapy in select cases. [Strong recommendation.]*

After progression on T-DXd, the weight of evidence supports tucatinib + trastuzumab + capecitabine as the preferred next option when not previously used, with T-DM1 reserved as an alternative and chemotherapy + a HER2-directed antibody as a fallback. The preference for tucatinib-based therapy is supported by randomized phase III data from HER2CLIMB [39,40], which showed that adding tucatinib to trastuzumab + capecitabine (vs. trastuzumab + capecitabine alone) significantly improved outcomes in a heavily pretreated HER2+ metastatic population with a median PFS 7.8 vs. 5.6 months and 1-year PFS 33.1% vs. 12.3% (HR for progression or death 0.54), along with a median OS of 21.9 months vs. 17.4 months in the overall study population.

In addition, there are now dedicated post-T-DXd cohorts showing that the tucatinib triplet retains meaningful activity after T-DXd exposure. In a 12-centre French cohort study of patients (*n* = 101) treated with tucatinib + trastuzumab + capecitabine after T-DXd, the overall response rate (ORR) was 32.6%, median PFS was 4.7 months (5.0 months when given immediately after T-DXd), and median OS was 13.4 months, with consistent benefit whether or not baseline brain metastases were present [41].

T-DM1 remains a reasonable alternative for patients who have not yet received it or who are not candidates for tucatinib, based on the phase III EMILIA trial’s survival benefit over lapatinib/capecitabine (median OS 29.9 vs. 25.9 months; HR 0.75) and improved PFS (9.6 vs. 6.4 months; HR 0.65)—recognizing that these data predate T-DXd and that T-DM1’s performance specifically after T-DXd is supported mainly by smaller or indirect datasets [42]. In patients unable to receive these targeted options, chemotherapy + a HER2-directed antibody is an acceptable lower-evidence option, typically guided by tolerance, comorbidity, and patient preference.

**Recommendation 14:**

***For patients with HER2+ metastatic breast cancer whose disease has progressed after at least 2 HER2-directed therapies (one of which was an ADC)***, *the recommendation for treatment is tucatinib + capecitabine + trastuzumab (can be considered earlier if brain metastasis is present). [Strong recommendation.]*

Recommendation 14 is for patients who have progressed after T-DXd. See recommendation 13b HER2CLIMB.

**Figure 2:**
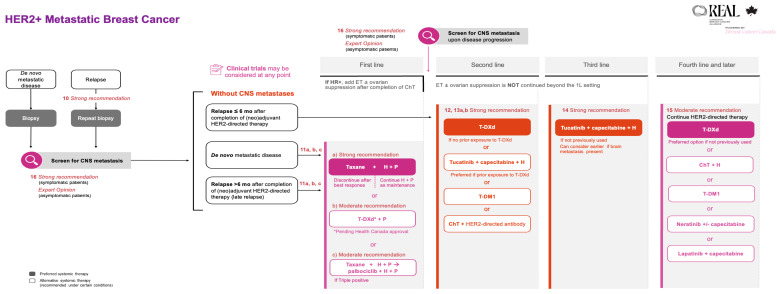
REAL Alliance recommendations for the treatment of metastatic breast cancer. 1L, first line; ChT, chemotherapy; CNS, central nervous system; ET, endocrine therapy; H, trastuzumab; HR+, hormone receptor positive; mo, months; P, pertuzumab; T-DM1, trastuzumab emtansine; T-DXd, trastuzumab deruxtecan.

All the REAL Alliance recommendations for metastatic breast cancer are outlined in an algorithm (see Figure 2).

### 3.3. Brain Metastases

REAL Alliance recommendations for patients with HER2+ metastatic breast cancer and brain metastases are summarized in Table 3 and compared with ESMO and ASCO guidelines. REAL Alliance recommendations are aligned with ESMO and ASCO guidelines where they exist. Importantly, REAL Alliance extends beyond current guidelines to address additional clinical situations, such as oligometastatic disease and sequencing of local and systemic therapies. These scenarios are frequently encountered in routine clinical practice yet are insufficiently addressed in existing guidelines and can pose significant management challenges. Other groups have also recognized this need and provided their guidance on real-world clinical situations for patients with HER2+ metastatic breast cancer and brain metastases [43].

Recommendations 22b and 25, and the supporting evidence, are revised in this 2025 update; see Table 3.

**Figure 3:**
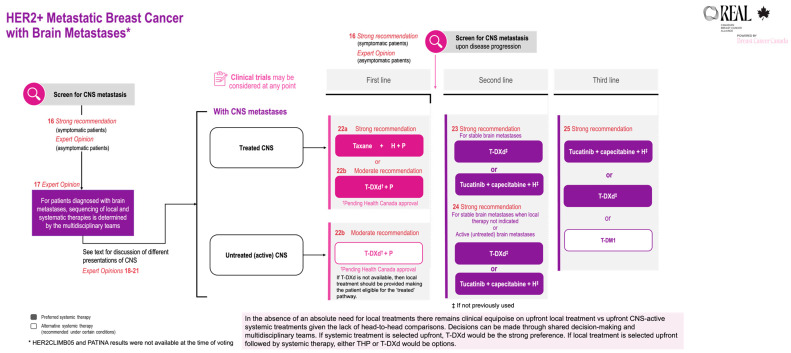
REAL Alliance recommendations for the treatment of brain metastases. CNS, central nervous system; H, trastuzumab; P, pertuzumab; T-DM1, trastuzumab emtansine; T-DXd, trastuzumab deruxtecan; THP, taxane + trastuzumab + pertuzumab.

The REAL Alliance recommendations for brain metastases are outlined in Figure 3.

**Recommendation 22b:**

For patients with HER2+ metastatic breast cancer with treated or active (untreated) brain metastases, T-DXd * + pertuzumab is the preferred systemic option in the first-line metastatic setting. [Moderate recommendation.]

* Pending Health Canada approval

Although CLEOPATRA is an established first-line regimen for patients with HER2+ metastatic breast cancer, data regarding its intracranial efficacy is lacking [33]; in this trial, the addition of pertuzumab to TH slightly delayed the onset of CNS metastases by 3.1 months but the incidence of CNS metastases was nearly identical at the time of trial completion in both arms of the study [45].

T-DXd has since transformed expectations for systemic efficacy in the presence of brain metastases [46]. In a pooled CNS analysis of DESTINY-Breast 01/02/03 [47], the median intracranial duration of response was 12.3 months (95% CI 9.1–17.9) for treated/stable and 17.5 months (95% CI 13.6–31.6) for untreated/active CNS metastases, with a corresponding median CNS-PFS of 12.3 months (95% CI 11.1–13.8) and 18.5 months (95% CI 13.6–23.3), respectively. DESTINY-Breast09 demonstrated that T-DXd + pertuzumab resulted in a significant median PFS benefit compared to THP in the first-line treatment of patients with HER2+ metastatic breast cancer [38]. Though only 6% of the study population had CNS metastases, the median PFS in that subgroup was 31.8 months in the T-DXd + pertuzumab arm compared to 9.5 months in the THP arm (HR 0.30) [38].

The phase IIIb/IV DESTINY-Breast12 study [48] further confirmed these findings in a dedicated brain metastasis cohort (*n* = 263): the 12-month PFS rate was 61.6% (95% CI 54.9–67.6), median PFS was 17.3 months (95% CI 13.7–22.1) and CNS-ORR was 71.7% (95% CI 64.2–79.3) among patients with measurable brain metastases. Although most patients were enrolled in DESTINY-Breast12 after 1 or 2 lines of prior systemic therapy, those with baseline brain metastases in the first-line setting were also represented (7.6%). Results of the TUXEDO-1 trial [49], DEBBRAH trial [50], and a recent systematic review and meta-analysis (10 studies; *n* = 319) [51] also corroborated these findings. Collectively, these data support T-DXd as a HER2-directed ADC with robust and durable CNS efficacy in HER2+ metastatic brain cancer.

In patients without an urgent need for local CNS intervention, there is clinical equipoise between upfront local therapy and upfront CNS-active systemic therapy due to the absence of comparative sequencing data. Decisions should be individualized through shared decision-making and multidisciplinary review. Both strategies—initial systemic therapy with deferred radiation or surgery, or upfront local therapy followed by systemic treatment—are acceptable. When systemic therapy is initiated first, T-DXd is preferred. Following local therapy, either THP or T-DXd may be considered, with a slight preference for T-DXd based on its intracranial efficacy. Until such time as Health Canada approval is granted, clinicians should continue to use approved regimens consistent with current standards of care.

For patients with stable extracranial disease and intracranial progression, which is not an uncommon scenario for patients with HER2+ metastatic breast cancer [52], considerations include continuation of current systemic therapy with local treatment intervention versus a switch to a CNS-active systemic regimen if available and feasible. The single-arm, phase II BRIDGET/BRE21-516 trial [NCT05323955] is evaluating the possible benefit of adding tucatinib to HP or T-DM1 among patients with isolated intracranial progression who have received local treatment intervention if indicated. A similar single-arm trial among patients with isolated brain progression after local therapy is ongoing in France [NCT05041842, “InTTercePT”]. However, currently, it is unclear whether a switch in systemic therapy provides benefit above and beyond local treatment intervention in this scenario. Multidisciplinary tumour-board review is essential to tailor/sequence local and systemic therapies, monitor toxicity, and optimise durable CNS disease control and patient quality of life.

**Recommendation 25:**

*For patients with HER2+ metastatic breast cancer and active brain metastases whose disease has progressed after prior multiple systemic therapies, subsequent treatment should be individualized according to prior HER2-directed treatment exposure, as follows:*

*(a)*  *If T-DXd has not been received: either T-DXd or tucatinib + capecitabine + trastuzumab are evidence-based options. [Strong recommendation.]*
*(b)*  *If T-DXd has already been used: tucatinib + capecitabine + trastuzumab (preferred) or T-DM1 may be considered. [Strong recommendation.]*

Recommendation 22b outlines the place in therapy of T-DXd for patients with CNS metastases. Thus, for patients with HER2+ breast cancer and active brain metastases whose disease has progressed after multiple treatments and who have not yet received T-DXd, either T-DXd or the HER2CLIMB regimen would be preferred treatment options. Statements 23 and 24, as outlined in Table 3 and reviewed in our 2024 publication, refer to tucatinib + capecitabine + trastuzumab as a treatment option based on the randomized HER2CLIMB trial, which established tucatinib + capecitabine + trastuzumab as another cornerstone regimen for patients with HER2+ metastatic disease (including those with brain metastases). Among 291 patients with baseline brain metastases, tucatinib reduced the risk of intracranial progression or death by 68% (HR 0.32, *p* < 0.001) and extended median CNS-PFS from 4.2 to 9.9 months, with a median OS gain of 9.1 months [39]. These data support tucatinib as an effective option for patients with active CNS disease in the second- or third-line metastatic setting; given that a head-to-head comparison between T-DXd and HER2CLIMB regimens is lacking, the sequence of therapies depends on prior exposure and expected treatment tolerance.

While T-DXd and tucatinib + capecitabine + trastuzumab represent the most active systemic options for patients with HER2+ metastatic breast cancer and progressive CNS disease, T-DM1 retains a role for patients who have exhausted or are ineligible for T-DXd or tucatinib-based therapy. In the KAMILLA real-world cohort, the intracranial ORR was ~20%, with a median PFS of 5–6 months [53], substantially lower than for T-DXd or tucatinib-based therapy but offering a well-tolerated option in selected patients. Evidence for chemotherapy + HER2-directed antibody combinations in the CNS setting remains limited and largely extrapolated from extracranial outcomes. However, these approaches may still provide meaningful disease control in appropriately selected patients.

REAL Alliance recommends that treatment choice in this setting be guided by prior regimen exposure, disease biology, anticipated toxicities, patient preferences, and quality-of-life considerations. Multidisciplinary discussions are essential.

## 4. Limitations

This document represents expert consensus guidance rather than a formal systematic review. Although sub-committee members reviewed peer-reviewed publications and major conference presentations with potential practice-changing implications, the evidence selection process was not conducted using a predefined systematic search strategy.

The oncology treatment landscape is rapidly evolving, and some recommendations are informed by recently presented data with immature overall survival follow-up. As such, ongoing updates will be required as additional data emerge.

Clinical trial populations may not fully reflect the diversity and comorbidity burden of patients seen in routine practice, including older adults and those with significant organ dysfunction. Treatment decisions in these populations require individualized clinical judgment.

Finally, while recommendations are based on the best available evidence and developed independent of reimbursement considerations, variation in regulatory approval timing and provincial implementation may influence real-world adoption.

## 5. Conclusions

The treatment landscape for HER2+ breast cancer continues to evolve rapidly, with practice-changing data emerging regularly at international congresses. REAL Alliance is committed to providing yearly updates to our consensus recommendations to support consistent, high-quality, and equitable care for patients with HER2+ breast cancer across Canada.

## Figures and Tables

**Table 1:** Summary of REAL Alliance recommendations for the treatment of HER2+ early breast cancer and comparison with those from ESMO and ASCO.

Recommendations for Early Breast Cancer (Recommendations 1–4, 6, 7, 9 Reprinted from Manna 2024 [1] with Permission)		REAL	ESMO [10]	ASCO [11]
**1**	*cT1a and b, cN0* **For patients with HER2+ early breast cancer cT1a and b (i.e., ≤1 cm) without evidence of nodal disease (cN0)**, the standard of care is timely surgery followed by adjuvant treatment depending on the pathologic staging of disease (*see Recommendations 3–4)*.	Strong recommendation ●●●	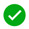	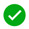
**2** **Narrative updated for 2025**	*cT1c, cN0* **For patients with HER2+ early breast cancer cT1c (i.e., >1 to ≤2 cm) without evidence of nodal disease (cN0)**: (a) The standard of care is surgery followed by adjuvant treatment. (b) However, due to current global practice, consideration can be given to neoadjuvant treatment followed by surgery and adjuvant treatment.	Strong recommendation ●●● Moderate recommendation ●●	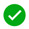 ESMO does not include this statement	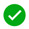 ASCO does not include this statement
**3**	*pT1, pN0* **For patients with HER2+ early breast cancer with pT1 without evidence of nodal disease (pN0)**, the standard of care adjuvant systemic treatment is paclitaxel + trastuzumab for 12 weeks, followed by trastuzumab monotherapy for 9 months.	Strong recommendation ●●●	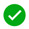	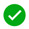
**4**	*≥pT2, pN0* **Although neoadjuvant treatment is preferred, for those patients who are treated with upfront surgery and are *then* found to have ≥pT2 pN0 disease,** the standard of care is adjuvant chemotherapy + trastuzumab.	Strong recommendation ●●●	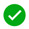	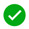
**5** **Recommendation updated in 2025**	*≥cT2 or cN+* **For patients with HER2+ early breast cancer with ≥cT2 or those with nodal disease (cN+)**, the standard of care is neoadjuvant therapy. Treatment options are: (a) Trastuzumab + pertuzumab + chemotherapy (taxane backbone preferred); (b) For those meeting DESTINY-Breast11 criteria *, T-DXd *^†^* followed by trastuzumab + pertuzumab + taxane. * cT3 and cN0–3 or cT and cN1–3 *^†^* Pending Health Canada approval	Strong recommendation ●●● Moderate recommendation ●●	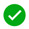 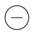 ESMO not yet updated with DB11	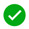 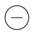 ASCO not yet updated with DB11
**6** **Narrative updated in 2025**	*pN+* **Although neoadjuvant treatment is preferred, for those patients who are treated with upfront surgery and are *then* found to have nodal disease in the pathological specimen (pN+),** the standard of care is adjuvant trastuzumab + chemotherapy followed by trastuzumab, with consideration given to the addition of pertuzumab for a total of 1 year.	Moderate recommendation ●●	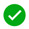	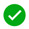
**7**	*Pathologic complete response* **For patients with HER2+ early breast cancer in whom a pathologic complete response is determined in the surgical specimen after completion of neoadjuvant trastuzumab + pertuzumab + chemotherapy**, the standard of care is trastuzumab for a total of 1 year.	Strong recommendation ●●●	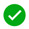 If cN0 at initial diagnosis If cN+ at initial diagnosis, then HP	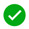
**8** **Recommendation updated in 2025**	*Residual invasive disease* **For patients with HER2+ early breast cancer in whom residual invasive disease is detected pathologically in the surgical specimen of the breast or axillary lymph nodes after completion of neoadjuvant trastuzumab + pertuzumab + chemotherapy, the standard of care is** **escalation of adjuvant therapy**. Treatment options include: (a) For disease not meeting high-risk criteria *, adjuvant therapy with T-DM1 for 14 cycles. (b) For high-risk disease*, adjuvant therapy with T-DXd *^†^* for 14 cycles, replacing T-DM1. * High-risk criteria: inoperable early breast cancer (cT4, N0–3 or cT1–3, N2–3) or operable early breast cancer (cT1–3, N0–1) with axillary node-positive disease (ypN1–3) after neoadjuvant chemotherapy. *^†^* Pending Health Canada approval	Strong recommendation ●●● Strong recommendation ●●●	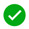 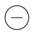 ESMO not yet updated with DB05	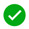 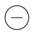 ASCO not yet updated with DB05
**9**	*Hormone receptor-positive disease* **Despite the lack of survival benefit, for patients with HER2+ HR+ and N+ disease who have completed (neo)adjuvant chemotherapy + trastuzumab**, extended adjuvant treatment with neratinib for 1 year after completion of trastuzumab-based adjuvant therapy can be considered to decrease recurrence.	Moderate recommendation ●●	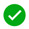	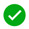

●●●, strong recommendation; ●●, moderate recommendation; 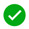, alignment; 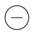, some variation. Purple text describes the tumour and nodal status, and bolded text describes the patient.

**Table 2:** Summary of REAL Alliance recommendations for the treatment of HER2+ metastatic breast cancer and comparison with those from ESMO and ASCO.

Recommendations for Metastatic Breast Cancer (Recommendations 10, 12, 15 Reprinted from Manna 2024 [1] with Permission)		REAL	ESMO [28,29]	ASCO [30,31]
**10**	*Repeat biopsy* When safe and feasible, repeat biopsy should be performed in **all patients whose disease relapses on or after adjuvant treatment**.	Strong recommendation ●●●	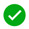	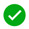
**11** **Recommendation updated in 2025**	*1L treatment de novo disease or late relapse* **For patients with HER2+ (HR±) metastatic breast cancer:** **Who have not received prior HER2-directed therapy or chemotherapy (de novo);** **Whose disease relapses >6 months after completion of (neo)adjuvant chemotherapy + HER2-directed therapy (late relapse),** treatment options include: (a) Trastuzumab + pertuzumab + taxane chemotherapy, followed by trastuzumab + pertuzumab +/− ET maintenance therapy, the current standard of care. **(b)** T-DXd * + pertuzumab could be considered using shared decision-making. * Pending Health Canada approval **(c) For triple-positive disease only,** trastuzumab + pertuzumab + taxane chemotherapy, followed by maintenance palbociclib + ET (aromatase inhibitor or fulvestrant ± ovarian suppression) + trastuzumab + pertuzumab, could be considered.	Strong recommendation ●●● Moderate recommendation ●● Moderate recommendation ●●	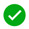 NC 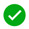	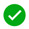 NC NC
**12**	*1L treatment early relapse* **For patients with HER2+ (HR±) metastatic breast cancer whose disease relapses ≤6 months after completion of (neo)adjuvant chemotherapy + HER2-directed therapy**, the recommended treatment is as per the second-line recommendation (*see Recommendation 13*).	Strong recommendation ●●●	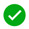 (relapse ≤ 12 mos)	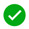
**13** **Recommendation updated in 2025**	*2L treatment* **(a) For patients with HER2+ metastatic breast cancer whose disease has progressed on first-line HER2-directed therapy that was not T-DXd**, the standard of care is T-DXd, in the absence of contraindications. (b) **For patients with HER2+ metastatic breast cancer whose disease has progressed following T-DXd,** treatment options include tucatinib + capecitabine + trastuzumab (preferred), T-DM1, or chemotherapy + HER2-directed antibody therapy in select cases.	Strong recommendation ●●● Strong recommendation ●●●	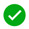 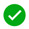	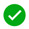 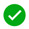
**14** **Recommendation updated in 2025**	*3L treatment* **For patients with HER2+ metastatic breast cancer whose disease has progressed after at least 2 HER2-directed therapies (one of which was an ADC),** the recommendation for treatment is tucatinib + capecitabine + trastuzumab (can be considered earlier if brain metastases are present).	Strong recommendation ●●●	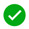	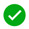
**15**	*4L and later treatment* **For patients with HER2+ metastatic breast cancer whose disease has progressed after at least 3 HER2-directed therapies**, the recommendation based on evidence is to continue HER2-directed therapy. Options are: chemotherapy + trastuzumab or another monoclonal antibody; T-DM1; neratinib +/− capecitabine; and lapatinib + capecitabine.	Moderate recommendation ●●	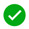	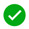

●●●, strong recommendation; ●●, moderate recommendation; 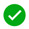, alignment; NC, not covered by ESMO or ASCO. Purple text describes the tumour and nodal status, and bolded text describes the patient.

**Table 3:** Summary of REAL Alliance recommendations for the treatment of HER2+ metastatic breast cancer with brain metastases and comparison with those from ESMO and ASCO.

Recommendations for Brain Metastases (Recommendations 16–21, 23, and 24 are Reprinted from Manna 2024 [1] with Permission)		REAL	ESMO [28,29]	ASCO [44]
**16**	*CNS screening* **For patients with HER2+ metastatic breast cancer who have symptoms suggestive of CNS metastases**, appropriate diagnostic investigations for CNS metastases are essential. **For patients with HER2+ metastatic breast cancer,** screening for CNS metastases should be considered in **asymptomatic patients** at baseline in the metastatic setting and at disease progression.	Strong recommendation ●●● REAL Alliance expert opinion ○	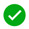 If detection of CNS metastases will alter the choice of systemic therapy	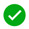 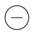
**17**	*Multidisciplinary care* **For patients with a history of HER2+ metastatic breast cancer who are diagnosed with brain metastases**, multidisciplinary care with representation from radiology, radiation oncology, neurosurgery, medical oncology, and supportive care is the standard of care, with the multidisciplinary team providing recommendations on sequencing of local and systemic therapies.	REAL Alliance expert opinion ○	NC	NC
**18**	*Characteristics of CNS disease at screening* **For patients with a history of HER2+ metastatic breast cancer but without other extracranial systemic disease who present with oligometastatic brain metastases amenable to local therapy**, there is insufficient evidence to make a recommendation for a change in systemic therapy. Multidisciplinary care is the standard of care, and the multidisciplinary team is to make recommendations on the sequencing of local and systemic therapies in such patients.	REAL Alliance expert opinion ○	NC	NC
**19**	*Characteristics of CNS disease at screening* **For patients with a history of HER2+ metastatic disease who present with asymptomatic, low-volume, newly diagnosed brain metastases**, treatment should be discussed by MDT incorporating patient values with treatment options, including initial HER2-directed systemic therapy versus upfront local therapy.	REAL Alliance expert opinion ○	NC	NC
**20**	*Characteristics of CNS disease at screening* **For patients with a history of HER2+ metastatic disease who present with symptomatic, newly diagnosed brain metastases**, upfront stereotactic radiosurgery is a reasonable approach when technically feasible (and often preferred over whole brain radiotherapy).	REAL Alliance expert opinion ○	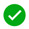	NC
**21**	*Characteristics of CNS disease at screening* **For patients with HER2+ metastatic breast cancer with parenchymal CNS disease**, the decision to offer systemic therapy prior to local therapies should be individualized for each patient and ideally discussed at multidisciplinary rounds. Key considerations include tumour burden and clinical symptoms. A multidisciplinary approach should be conducted to confirm if and when systemic therapy should be held during local CNS therapy to reduce the risk of toxicities and radiation necrosis (for patients receiving an ADC).	REAL Alliance expert opinion ○	NC	NC
**22** **Recommendation updated in 2025**	*1L treatment brain metastases* **(a) For patients with HER2+ metastatic breast cancer with active or progressive systemic disease in the presence of treated brain metastases**, the current standard of care in the first-line setting is trastuzumab + pertuzumab + taxane. **(b) For patients with HER2+ metastatic breast cancer with treated or active (untreated) brain metastases**, T-DXd* + pertuzumab is the preferred systemic option in the first-line metastatic setting. * Pending Health Canada approval	Strong recommendation ●●● Moderate recommendation ●●	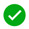 NC	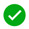 NC
**23**	*2L treatment stable brain metastases* **For patients with HER2+ metastatic breast cancer with stable brain metastases whose disease has progressed on first-line therapy**, the standard of care options are T-DXd or tucatinib + capecitabine + trastuzumab.	Strong recommendation ●●●	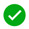	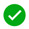
**24**	*2L treatment active brain metastases* **For patients with HER2+ metastatic breast cancer and asymptomatic active (i.e., untreated) or stable brain metastases where local therapy is not indicated and whose disease has progressed on first-line therapy**, options include tucatinib + capecitabine + trastuzumab or T-DXd (if not previously used). Such cases should be reviewed by the multidisciplinary team to determine the sequencing of local and systemic therapies.	Strong recommendation ●●●	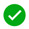	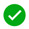
**25** **Recommendation updated in 2025**	*3L treatment* **For patients with HER2+ metastatic breast cancer and active brain metastases whose disease has progressed after prior multiple systemic therapies, subsequent treatment should be individualised according to prior HER2-directed exposure, as follows:** (a) If T-DXd has not been received: either T-DXd or tucatinib + capecitabine + trastuzumab are evidence-based options. (b) If T-DXd has already been used, tucatinib + capecitabine + trastuzumab (preferred) or T-DM1 may be considered.	Strong recommendation ●●● Strong recommendation ●●●	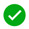 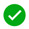	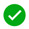 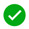

●●●, strong recommendation; ●●, moderate recommendation; ○, REAL Alliance expert opinion. 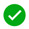, alignment; 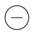, some variation; NC, not covered by ESMO or ASCO. Purple text describes the tumour and nodal status, and bolded text describes the patient.

## Data Availability

No new data was generated.

## Supplementary Materials

The following supporting information can be downloaded at: https://www.mdpi.com/article/10.3390/curroncol33040200/s1, Table S1: voting summary.

## Abbreviations

The following abbreviations are used in this manuscript:

1L	First line
2L	Second line
3L	Third line
4L	Fourth line
AC	Anthracycline + cyclophosphamide
ADC	Antibody–drug conjugate
ASCO	American Society of Clinical Oncology
CDK4/6	Cyclin-dependent kinase 4 and 6
CI	Confidence interval
CNS	Central nervous system
DB05	DESTINY-Breast05
DB09	DESTINY-Breast09
DB11	DESTINY-Breast11
ddAC	Dose-dense doxorubicin + cyclophosphamide
DFS	Disease-free survival
EFS	Event-free survival
ESMO	European Society for Medical Oncology
ET	Endocrine therapy
HER2+	Human epidermal growth factor receptor 2-positive
HP	Trastuzumab + pertuzumab
HR+	Hormone receptor positive
ILD	Interstitial lung disease
MDT	Multidisciplinary team
ORR	Overall response rate
OS	Overall survival
pCR	Pathologic complete response
PFS	Progression-free survival
REAL Alliance	Research Excellence, Active Leadership Canadian Breast Cancer Alliance
SABCS	San Antonio Breast Cancer Symposium
T-DM1	Trastuzumab emtansine
T-DXd	Trastuzumab deruxtecan
TCH	Taxane + carboplatin + trastuzumab
TCHP	Taxane + carboplatin + trastuzumab + pertuzumab
TH	Taxane + trastuzumab
THP	Taxane + trastuzumab + pertuzumab
