# Supplementary material for: Guidance for Canadian Breast Cancer Practice: National Consensus Recommendations for the Systemic Treatment of Patients with HER2+ Breast Cancer in Both the Early and Metastatic Settings (2025 Update)
Source: Curr Oncol. 2026 Mar 31;33(4):200. doi: 10.3390/curroncol33040200 (PMC13115210; doi:10.3390/curroncol33040200)
Supplement: Supplementary file 1 [file curroncol-33-00200-s001.zip › curroncol-4128119-supplementary.pdf]

Voting Results

National Consensus Recommendations for HER2+

Voting results (Agree with statement as is, Agree with statement with edits, Do not agree with statement, Abstain)

		Consensus Recommendation	Consensus was reached on
5.	*≥cT2 or cN+*<br><br>**For patients with HER2+ early breast cancer with ≥cT2 or those with nodal disease (cN+),** the standard of care is neoadjuvant therapy. Treatment options are:<br>a) Trastuzumab + pertuzumab + chemotherapy (taxane preferred) [Strong recommendation], or<br>b) *T-DXd followed by trastuzumab + pertuzumab + taxane. [Moderate recommendation]	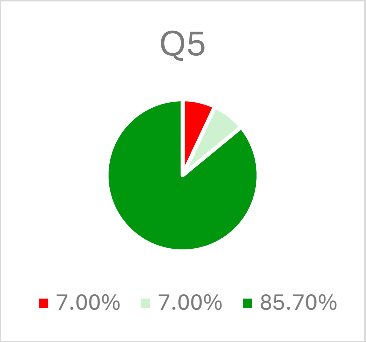<br>1 abstained<br>N=15	2nd round
8.	*Residual invasive disease*<br><br>**For patients with HER2+ early breast cancer in whom residual invasive disease is detected pathologically in the surgical specimen of the breast or axillary lymph nodes after completion of neoadjuvant trastuzumab + pertuzumab + chemotherapy,** the standard of care:<br>a) For high-risk disease* is adjuvant therapy with **T-DXd for 14 cycles, replacing T-DM1<br>b) For disease not meeting high-risk criteria is adjuvant therapy with T-DM1 for 14 cycles.	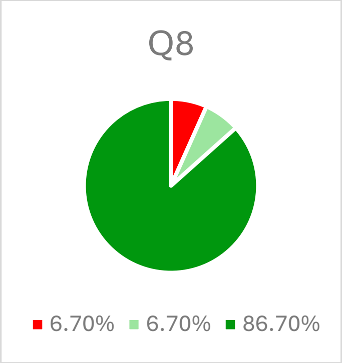<br>N=15	2nd round

11. b)	**For patients with de novo HER2-positive (HR±) metastatic breast cancer who have not received prior HER2-directed therapy or chemotherapy for metastatic disease, OR for those with disease recurrence occurring >6 months after completion of (neo)adjuvant chemotherapy + HER2-directed therapy**, *T-DXd + pertuzumab could be considered with shared decision-making. (Moderate recommendation)<br><br>*Subject to Health Canada approval	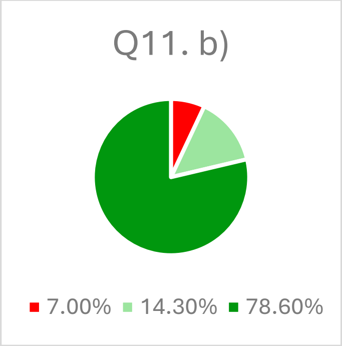<br>1 abstained<br>N=15	2nd round
11. c)	**For patients with de novo HER2-positive, hormone receptor–positive (triple-positive) metastatic breast cancer who have not received prior HER2-directed therapy or chemotherapy for metastatic disease, OR for those with disease recurrence occurring >6 months after completion of (neo) adjuvant chemotherapy + HER2-directed therapy**, trastuzumab + pertuzumab + taxane chemotherapy followed by maintenance palbociclib + endocrine therapy (aromatase inhibitor or fulvestrant ± ovarian suppression) + trastuzumab + pertuzumab may be considered. [Strong recommendation]	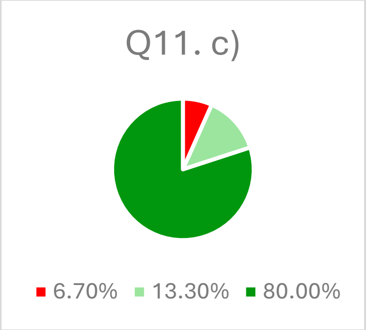<br>N=15	2nd round
13. b)	**For patients with HER2-positive metastatic breast cancer whose disease has progressed following T-DXd,** treatment options include tucatinib + capecitabine + trastuzumab (preferred if not previously used), or trastuzumab emtansine (T-DM1), or chemotherapy + HER2-directed antibody therapy in select cases. [Strong recommendation]	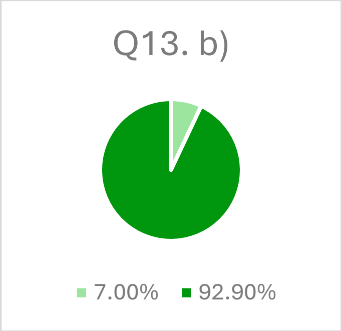<br>1 abstained<br>N=15	2nd round

22. b)	**For patients with HER2-positive metastatic breast cancer with treated or active,** untreated brain metastases, *T-DXd may be considered as a systemic option based on emerging evidence of intracranial activity.<br><br>* Subject to Health Canada approval [moderate consideration]	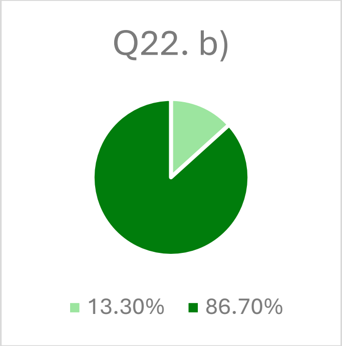<br>N=15	2nd round
25.	*3L treatment*<br><br>For patients with HER2-positive metastatic breast cancer and active brain metastases whose disease has progressed after prior multiple systemic therapies, subsequent treatment should be individualized according to prior HER2-directed exposure:<br>○ If T-DXd has not been received: either T-DXd (preferred) or tucatinib + capecitabine + trastuzumab are evidence-based options.<br>○ If T-DXd has already been used: tucatinib + capecitabine + trastuzumab (preferred, if not previously given) or trastuzumab emtansine (T-DM1) or chemotherapy + HER2-directed antibody may be considered. [Strong recommendation]	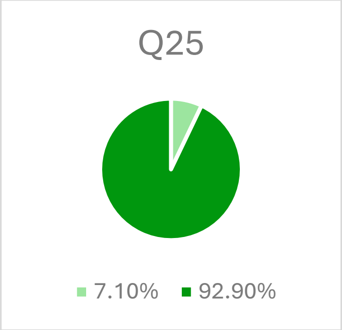<br>1 abstained<br>N=15	2nd round
